# 肺上皮样血管内皮瘤的研究进展

**DOI:** 10.3779/j.issn.1009-3419.2019.07.10

**Published:** 2019-07-20

**Authors:** 航 林, 远大 程, 春芳 张

**Affiliations:** 410008 长沙，中南大学湘雅医院胸外科 Department of Thoracic Surgery, Xiangya Hospital, Central South University, Changsha 410008, China

**Keywords:** 上皮样血管内皮瘤, 肺上皮样血管内皮瘤, 发病机制, 诊断, 治疗, 预后, Epithelioid hemangioendothelioma, Pulmonary epithelioid hemangioendothelioma, Etiopathogenesis, Diagnosis, Treatment, Prognosis

## Abstract

上皮样血管内皮瘤（epithelioid hemangioendothelioma, EHE）是一种罕见的血管肿瘤，起源于血管内皮细胞，可发生于身体多个部位，以软组织（四肢）、骨骼、肝脏和肺为主。相比肺部其他肿瘤而言，肺上皮样血管内皮瘤（pulmonary epithelioid hemangioendothelioma, P-EHE）比较少见。国外报道约百例，国内有数十例报道。由于P-EHE发病率低、临床症状及影像学表现缺乏特异性，因此经常被误诊或漏诊且未接受适当治疗，从而导致其预后不良。目前，组织学及免疫组织化学检查是诊断该病的重要依据。鉴于P-EHE较为少见，尚无统一的治疗方案。若病灶小且结节数量有限，手术是首选的治疗方法，同时达到诊断和治疗的目的。本文现就有关P-EHE的发病机制、临床表现、诊断、治疗方法、预后予以综述。

## Abstract (en)

Epithelioid hemangioendothelioma (EHE) is a rare vascular neoplasm that develops from vascular endothelial cells. It has been reported to occur many sites of body, but the most common EHE presentations are soft tissue (limbs), bone, liver and lung. Compared with other pulmonary tumors, pulmonary epithelioid hemangioendothelioma (P-EHE) is relatively rare. According to a literature review, more than 100 cases have been described all over the world. Due to the low incidence of P-EHE, lack of specificity in clinical symptoms and radiological findings, it is often misdiagnosed. Meanwhile, many patients do not receive appropriate treatment, resulting in poor prognosis in some cases. Histology and immunohistochemical methods are essential for diagnosis. However, there is no established standard treatment for P-EHE, because of the rarity of the disease. When the lesions are small and limited in number, surgical is the best treatment, achieving the purpose of diagnosis and treatment at the same time. This article tries to present the etiopathogenesis, clinical manifestations, diagnosis, treatment and prognosis of P-EHE.

## 1 背景

上皮样血管内皮瘤（epithelioid hemangioendothelioma, EHE）是一种罕见的血管肿瘤，起源于血管内皮细胞^[1]^。其发病率不足百万分之一，占所有血管肿瘤的不到1%^[2]^。该病可发生于身体多个部位，以软组织（四肢）、骨骼、肝脏和肺为主^[2]^。由于EHE目前较为少见，国内外文献报道均以个案和小样本病例为主。

1975年，Dail和Liebow首次报道该病^[3]^。最初，由于病灶侵犯邻近的血管和小气道，故称其为血管内细支气管肺泡瘤（intravascular bronchioloalveolar tumor, IVBAT）^[3]^。1978年，Corrin等^[4]^通过免疫组织化学技术证实了肿瘤细胞是从内皮细胞系分化而来。1982年，Weiss和Enzinger将IVBAT更名为EHE^[5]^，用来描述受累于骨骼和软组织的血管肿瘤，其恶性程度介于完全良性的血管瘤和高度恶性的血管肉瘤之间^[5]^。2015年，世界卫生组织肺部肿瘤分类明确将EHE归类为低级别至中等级别恶性血管肿瘤，具有潜在转移能力^[6]^。

肺上皮样血管内皮瘤（pulmonary epithelioid hemangioendothelioma, P-EHE）属于间叶性肿瘤^[6]^。相比肺部其他肿瘤而言，P-EHE比较少见，具有一定的研究意义。目前关于P-EHE的研究中，约80%为女性患者^[7]^，发病年龄为40岁^[7]^，而胸膜EHE在男性患者中更常见^[8, 9]^。该病通常好发于中青年患者^[10]^，但也报道过儿童和老年人的病例。受累年龄范围较广，从7岁到83岁不等^[11, 12]^，发病年龄为36岁^[13]^。

## 2 发病机制

EHE的发病机制仍未明确。近几年，关于EHE的细胞分子遗传学研究有了突破性的进展。在分子水平上，不同的血管生成刺激因子可以作为内皮细胞增殖的启动子。有报道称，EHE的发生需要通过单核细胞趋化蛋白-1刺激内皮细胞的血管生成而促进肿瘤的发展^[14]^。Tanas等^[15, 16]^在大多数EHE中注意到t（1;3）（p36;q25）易位，而这种染色体易位在其他任何血管肿瘤中均未发现，如上皮样血管瘤、上皮样血管肉瘤或假性肌源性血管内皮瘤等。他们从已知t（1;3）（p36;q25）易位的肿瘤样本中分离出总RNA，然后构建了一个cDNA文库。通过配对末端全转录组测序（RNA-seq）对cDNA文库进行深度测序，并使用FusionSeq对其进行分析。最终，他们发现了一个mRNA融合转录物，其包含了位于3p25的*WWTR1*和位于1p36的*CAMTA1*^[15]^。值得注意的是，融合转录物不在相邻的正常组织中表达^[15]^。通过对其进行逆转录-聚合酶链反应（reverse transcription-polymerase chain reaction, RT-PCR）分析，结果证实了*WWTR1*与*CAMTA1*发生融合^[15]^。上述两个基因*WWTR1*和*CAMTA1*，前者编码转录辅助激活蛋白，即一种在内皮细胞中高度表达的蛋白；后者则编码转录调控蛋白，即一种通常只在大脑中表达的蛋白^[17]^。这两种基因发生融合是在*WWTR1*启动子的转录控制下，激活CAMTA1异常表达，从而使*WWTR1*的氨基末端与*CAMTA1*的羧基末端相互连接，编码出一种特定的融合转录因子^[15]^。所以，在肿瘤发生过程中，这两个基因发挥着重要的作用。另外，Antonescu等^[18]^在对EHE进行WWTR1-CAMTA1基因融合筛选过程中，发现了一组*YAP1-TFE3*基因融合的亚群。该亚群表现出独特的形态学特征，如血管形成良好，成熟的管腔内有上皮样细胞，嗜酸性胞浆丰富，主要出现在年轻患者身上^[16, 18]^。

最近，关于EHE发病机制的另一种推测认为慢性巴尔通体氏杆菌感染与这种恶性血管肿瘤的发生有因果关系。巴尔通体氏杆菌侵入人体后，会诱导红细胞和内皮细胞长期感染，包括至少三种巴尔通体氏杆菌（*B henselae*, *B Quintana*, *B bacilliformis*）诱导血管内皮生长因子，从而引起血管内皮细胞增殖^[19]^。巴尔通体氏杆菌是目前已知能引起内皮细胞增殖的唯一细菌属^[20]^。这些细菌病原体能上调有丝分裂和促炎基因，导致细胞骨架重组以及抑制内皮细胞凋亡。这表明这些细菌病原体可能有助于血管瘤的发展^[19]^。

## 3 临床表现

P-EHE缺乏典型的临床表现，通常表现为呼吸道症状（咳嗽、咳痰、呼吸困难等）伴胸痛，有少数患者会出现肺泡出血、咯血和贫血等不适^[21]^，偶尔也会出现杵状指和体质量减轻^[22]^。若累及胸膜时，常常伴有胸腔积液^[9]^。而近50%患者没有任何症状，大多数是在体检时偶然发现^[13, 23]^。这种罕见的血管肿瘤的临床表现会因肿瘤位置不同而不同。当EHE发生骨转移时，会有严重的病理性骨折的风险，如果发生在椎骨中，会导致脊柱受压，从而导致感觉异常、肌力减弱和截瘫^[24-26]^。

## 4 诊断

### 4.1 实验室检查

实验室检查缺乏特异性，血常规、血生化以及凝血功能基本正常，如果病灶涉及肝脏，血清碱性磷酸酶、天冬氨酸氨基转移酶、淀粉酶和脂肪酶可能会异常升高^[27]^。

### 4.2 影像学检查

影像学检查在术前诊断中具有一定价值。大多数P-EHE患者胸部CT表现为肺部多发结节灶^[28]^，主要分布在双肺下叶^[13]^，呈低密度。结节通常位于中小型血管和支气管附近，大小可达2 cm，但大多数直径小于1 cm^[13]^。有时会发展成孤立的肺结节，其大小可达5 cm^[22]^。同时，也可观察到肺门、纵隔淋巴结肿大，小叶间隔增厚和胸腔积液等^[29]^。而胸部增强CT检查可显示肿瘤全貌和血供之间的关系^[30]^。影像学上，钙化并不常见，但组织学检查常见坏死结节的钙化和骨化中心^[13]^。

对于P-EHE患者而言，^18^F-FDG-PET检查在评估肺结节葡萄糖代谢状况以及寻找原发病灶或其他转移灶方面具有重要价值^[31]^。Watanabe等^[32]^报道1例多发性肺结节的患者行^18^F-FDG-PET检查，静脉注射^18^F-FDG 1 h后仅有两个结节摄取增加，其标准化摄取值（standardized uptake value, SUV）分别为4.61和2.10。然后切除上述两个病灶，病理学检查证实为P-EHE。而其余未显示^18^F-FDG摄取增加的结节在术后随访过程中，大小无变化，保持稳定。因此，^18^F-FDG-PET检查在精确定位病灶的高代谢部位方面具有显著优势，而SUV则可作为制定P-EHE患者治疗方案的指标^[32]^。另外，其他文献[33-36]也报道了在EHE中^18^F-FDG摄取增加的案例，并肯定了^18^F-FDG-PET作为一种有效的无创成像方法，在评估EHE原发病灶或其他转移灶方面的潜在作用。

### 4.3 病理学特征

病理学依据是目前确诊EHE的唯一方法。肉眼可见，结节直径有数毫米到5 cm不等，切面呈灰白或黄棕色，质硬，无包膜^[23]^。镜下观，结节分带明显，结节周边细胞丰富，而结节中央则细胞稀少，同时伴有凝固性坏死、玻璃样变、淀粉样变，钙化甚至骨化^[22]^。肿瘤细胞为圆形、多角形，似上皮样形态，排列成小巢状、短索状、腺管状，呈舌乳头状、肾小球样增生，填充肺泡腔^[37]^。胞质丰富，嗜酸性或嗜双色性^[38]^，内有Webel-Palad小体、胞质内空泡以及基质软骨样、粘液样或透明样变性，是EHE的特征性结构，有助于EHE与上皮样血管肉瘤的鉴别^[39]^。细胞核呈圆形或卵圆形，核仁小或不明显，细胞异型性较低，染色质分布均匀，核分裂象少见^[37]^。偶尔可见纺锤状肿瘤细胞。这些细胞可形成大小不等的血管腔，腔内可见红细胞^[37]^。当肿瘤细胞出现异型性明显、病理性核分裂象≥2/10 HPF，且存在坏死灶时，则可诊断为中等级别EHE^[39]^。

肿瘤组织可侵犯小肺动脉、肺静脉和淋巴管^[23]^。在结节的外周区域，肿瘤细胞可通过Kohn孔浸润到邻近的肺泡，并以微息肉样的形式生长到细支气管内。然而，肺泡弹性框架基本上保持不变^[22]^。

免疫组织化学有助于EHE的诊断。CD34和CD31是公认的内皮细胞标志物，据报道，CD34在所有血管源性肿瘤中表达率超过90%，且在其他软组织肿瘤中也表达，因此在EHE的诊断中具有较差的特异性^[40]^。而CD31的表达局限于内皮细胞，巨噬细胞和血小板，相比之下，CD31则是相对特异的血管肿瘤标志物^[40]^。Fli-1蛋白是一种参与细胞增殖和肿瘤发生的核转录因子，其主要在内皮细胞、T细胞和巨核细胞中表达^[41]^。据报道，该蛋白可用于鉴别血管肿瘤的良恶性，并且显示出比内皮标志物CD31和CD34更好的敏感性和特异性^[40]^。Gill等^[40]^认为Fli-1和CD31是鉴别诊断的理想指标。同时，值得一提的内皮标记物为ERG，这是ETS家族中的转录因子，可在内皮细胞中表达，致癌基因*ERG*融合发生在前列腺癌，急性髓性白血病和尤文肉瘤中^[42-44]^。Miettinen等^[45]^报道，他们分析的EHE（42/43）、血管肉瘤（96/100）和卡波西肉瘤（26/26）的内膜上均有ERG表达。因此，ERG可以作为一种高特异性的良恶性血管肿瘤新标志物。此外，很少有EHE患者对细胞角蛋白表现出弱阳性，因此细胞角蛋白的表达对上皮起源的肿瘤可能没有特异性^[40]^。其他抗原，如波形蛋白、CK和EMA，也在P-EHE中部分表达^[46]^。迄今为止，EHE没有完全特异性肿瘤标志物，可通过组织学特征将其与其他上皮样血管瘤区分开来。

## 5 治疗方法

EHE的治疗方法各不相同，主要取决于肿瘤受累部位和程度，转移情况和个体因素。由于P-EHE较为少见，目前尚无统一的治疗方案。

### 5.1 随访观察

针对无任何症状的患者，经活检确诊为P-EHE后，每3个月-6个月定期随访观察可获得较好预后^[2, 3, 22]^。Kitaichi等^[22]^分析了亚洲21例P-EHE患者，其中3例无任何症状的P-EHE患者（1例男性和2例女性，均有双肺多发结节）均未接受任何治疗，在随访5年-15年期间内结节的数量和大小出现部分自行缓解。

### 5.2 手术治疗

若病灶小且结节数量有限，手术是首选的治疗方法，尤其对于单侧单发性或多发性结节的P-EHE患者而言，手术完全切除可达到最佳的治疗效果^[21]^。同时，Bagan等^[21]^发现肺楔形切除术与解剖性肺叶切除术具有相同的生存率。但目前由于发生淋巴结转移的患者较少，肺门、纵隔淋巴结清扫的预后价值仍不清楚^[21]^。然而，对于双侧多发性结节的患者也有选择手术治疗的个案报道。Eguchi等^[47]^报道了1例双肺多发性结节的P-EHE患者，对其进行双肺楔形切除术，共切除双侧32个肺结节，术后11年随访期间恢复较好，未见肿瘤复发。随着胸部微创技术的迅速发展，胸腔镜手术对该病的诊断及治疗较传统开胸手术有巨大的优势，尤其对于早期位置表浅、数量较少的肺结节往往能取到根治性效果^[48]^。

### 5.3 全身治疗

对于无法手术根治或出现广泛转移的患者，选择合适的辅助治疗方案非常关键。若患者的骨骼受累，可选择放疗，若病灶累及深部软组织或体内脏器，可优选化疗^[32]^。据文献报道，可用于P-EHE的化疗药物有卡铂、紫杉醇、阿霉素、长春瑞滨、吉西他滨等，其中卡铂联合紫杉醇是目前最常用的化疗方案^[49]^。但单独化疗的整体效果不明显^[22, 50]^。对于胸膜EHE患者而言，通常无法手术完全切除。Pinet等^[51]^报道了1例胸膜EHE患者，经卡铂联合依托泊苷治疗6个周期后病灶完全缓解。近年来，考虑到化疗药物在P-EHE中普遍无效，抗血管生成药物是治疗转移性P-EHE的一种有效方案。在P-EHE患者中，血管内皮生长因子（vascular endothelial growth factor, VEGF）高表达与肿瘤生长和转移有关^[52, 53]^。贝伐珠单抗（Bevacizumab）是一种针对VEGF的重组人源化单克隆抗体，已成功用于多种类型的恶性肿瘤^[54]^。目前，贝伐珠单抗联合卡铂、紫杉醇作为一线治疗方案，可使患者的病情得到部分缓解^[2]^。Belmont等^[55]^发现，P-EHE患者在经历顺铂联合依托泊苷治疗失败后，开始接受卡铂、紫杉醇联合贝伐珠单抗治疗。经5个周期治疗后，临床症状改善明显，病情得到部分缓解，并在13个月的随访过程中，情况稳定。叶波等^[56]^报道对1例P-EHE患者采用卡铂、紫杉醇及贝伐珠单抗联合治疗，该患者取得部分缓解，并随访存活15个月。Gaur等^[57]^也报道1例转移性EHE患者，其中肺与纵隔受累明显，在接受姑息性放疗后，开始给予贝伐珠单抗联合白蛋白结合型紫杉醇（nab-paclitaxel）治疗，在治疗3个月和6个月后，CT显示无新发转移灶，病情稳定。他们认为贝伐珠单抗联合白蛋白结合型紫杉醇对转移性P-EHE具有良好的耐受性和稳定性^[57]^。此外，沙利度胺^[58, 59]^、来那度胺^[60, 61]^、阿帕替尼^[49]^、帕唑帕尼^[62, 63]^、索拉非尼^[64, 65]^等多种药物都有临床应用的报道，也取得了不同程度的疗效，但多是个案报道或小样本临床研究，尚需更多的临床研究证实。

### 5.4 其他治疗

对于病灶局限某一器官且无法手术的患者而言，器官移植是一种有价值的治疗方法。Bonaccorsi-Riani等^[66]^报道目前肝上皮样血管内皮瘤已成为肝移植的良好适应证，接受肝移植治疗后5年和10年总生存率（overall survival, OS）分别为83%和74%，5年和10年无病生存率分别为82%和64%。因此，对于可能会出现呼吸衰竭的P-EHE患者，我们建议应该提倡肺移植作为P-EHE的替代治疗方法^[21]^。

## 6 预后

Lau等^[67]^回顾分析了206例EHE患者1年OS可达90%，5年OS为73%，而EHE出现进展后，1年OS则为53%，5年OS为24%，其中位生存期为1.3年。EHE中位进展时间为2.0年（范围0.3年-22.3年）^[67]^。同时，他们指出男性患者，年龄≥55岁，疾病进展是该病预后较差的不良因素^[67]^。Bagan等^[21]^对80例P-EHE患者进行回顾性分析，根据临床症状和影像学结果，将P-EHE患者分为两类：一类为有肺结节，但无症状患者中位生存期为180个月，而另一类为有肺结节，并出现明显症状的患者（例如：肺泡出血、咯血、胸腔积液、贫血）生存率明显较低，中位生存期不足1年。因此，他们认为体质量减轻、贫血、胸腔积液和肺部相关症状（呼吸困难、咳嗽、胸痛、咯血）是预后不良的重要因素^[21]^。另外，据文献^[2, 3, 22]^报道，若患者出现淋巴结及远处转移，肿瘤直径 > 3.0 cm，镜下见纺锤状肿瘤细胞，意味着预后更差。

对于双肺病灶伴有肺泡出血（间质性病变、毛玻璃样变）和血性胸腔积液的患者而言，呼吸衰竭通常是主要死亡的原因^[21]^。少数患者死于肿瘤的肺外转移，以肝、骨转移为主^[21]^。

## 7 展望

EHE是一种罕见的恶性血管肿瘤，其恶性程度介于完全良性的血管瘤和高度恶性的血管肉瘤之间。由于P-EHE发病率低、临床症状及影像学表现缺乏特异性，因此经常被误诊或漏诊且未接受适当治疗，从而导致其预后不良。因此明确其发病机制，制定标准的治疗方法尤为重要。现已发现该病的发生与*WWTR1-CAMTA1*基因融合，*YAP1-TFE3*基因融合以及慢性巴尔通体感染有关，因此，针对特定*WWTR1-CAMTA1*基因融合，*YAP1-TFE3*基因融合的靶向治疗可能在不久的将来产生巨大的影响。如果证实存在巴尔通体氏杆菌感染，那么根除细菌感染或阻断巴尔通体诱导血管生成和细胞增殖的信号可能会减缓肿瘤进展并改善患者预后。

表皮生长因子受体（epidermal growth factor receptor, EGFR）是上皮生长因子细胞增殖和信号传导的受体，属于ErbB受体家族的一种^[68]^。EGFR与肿瘤细胞增殖、血管生成、肿瘤侵袭、转移及细胞凋亡的抑制有关^[68]^。研究^[69]^表明在许多实体肿瘤中存在EGFR的高表达或突变，例如乳腺癌、肺癌、结直肠癌、食道癌、胶质瘤、肛门癌和胶质母细胞瘤。因此，P-EHE的血管形成、肿瘤进展可能也与EGFR过表达或突变有关，但目前缺乏相关研究。

总之，P-EHE比较少见，P-EHE的基础和临床均值得深入的研究和探讨，提高对P-EHE的认识，有助于P-EHE正确的诊断及早的治疗。
